# Supplementary material for: A UMLS-based spell checker for natural language processing in vaccine safety
Source: BMC Med Inform Decis Mak. 2007 Feb 12;7:3. doi: 10.1186/1472-6947-7-3 (PMC1805499; doi:10.1186/1472-6947-7-3)
Supplement: Additional file 13 — RAP application directory. Sets up directory for RAP (RDF application for PHP) source code [file 1472-6947-7-3-S13.gz › rap/doc/phpdoc/__filesource/fsource_resModel__resModelResAlt.php.html]

xml version="1.0" encoding="iso-8859-1"?


File Source for ResAlt.php


# Source for file ResAlt.php

Documentation is available at ResAlt.php

```
1. <?PHP
2. // ----------------------------------------------------------------------------------
3. // Class: ResAlt
4. // ----------------------------------------------------------------------------------


8. /**
9. * This interface defines methods for accessing RDF Alternative resources.
10. * These methods operate on the RDF statements contained in a model.
11. *
12. * <BR><BR>History:<UL>
13. * <LI>10-01-2004                : First version of this class.</LI>
14. *
15. * @version  V0.9.1
16. * @author Daniel Westphal <mail at d-westphal dot de>
17. *
18. * @package     resModel
19. * @access    public
20. ***/
21. class ResAlt extends ResContainer
22. {

24. /**
25. * Constructor
26. * You can supply a URI
27. *
28. * @param string $uri
29. * @access    public
30. */
31. function ResAlt($uri = null)
32. {
33. parent::ResContainer($uri);
34. $this->containerType=new ResResource(RDF_NAMESPACE_URI.RDF_ALT);
35. }

37. /**
38. *  Return the default value for this resource
39. *
40. * @return    object ResResource/ResLiteral
41. * @access    public
42. */
43. function getDefault()
44. {
45. //get the first memeber
46. $statements=$this->listProperties($this->_getMembershipPropertyWithIndex(1));
47. if(isset($statements[0]))
48. {
49. //return the value
50. return $statements[0]->getObject();
51. } else
52. {
53. return null;
54. }
55. }

57. /**
58. *  Set the default value of this container.
59. *
60. * @param    object ResResource/ResLiteral $object
61. * @access    public
62. */
63. function setDefault($object)
64. {
65. //remember the old default value
66. $oldDefaultObject=$this->getDefault();
67. //if there wasn't a default value before
68. if($oldDefaultObject === null)
69. {
70. //add the new value
71. $this->addProperty($this->_getMembershipPropertyWithIndex(1),$object);
72. } else
73. {
74. //remove the old value
75. $this->removeAll($this->_getMembershipPropertyWithIndex(1));
76. //set the new value
77. $this->addProperty($this->_getMembershipPropertyWithIndex(1),$object);
78. //add the old default value at the end
79. $this->add($oldDefaultObject);
80. }
81. }
82. }
83. ?>
```

Documentation generated on Thu, 7 Jul 2005 13:43:29 +0200 by phpDocumentor 1.3.0RC3
